# Research on the Natural Hazard Emergency Cooperation Behavior between Governments and Social Organizations Based on the Hybrid Mechanism of Incentive and Linkage in China

**DOI:** 10.3390/ijerph182413064

**Published:** 2021-12-10

**Authors:** Jida Liu, Changqi Dong, Shi An, Yanan Guo

**Affiliations:** 1 School of Management, Harbin Institute of Technology, Harbin 150001, China; kittadada@yeah.net (J.L.); 21B910041@stu.hit.edu.cn (C.D.); 2 Department of Engineering Systems and Services, Delft University of Technology, BX-2628 Delft, The Netherlands

**Keywords:** natural hazards, emergency cooperation, social organization, emergency interaction, evolutionary game theory

## Abstract

Social organizations have become an important component of the emergency management system by virtue of their heterogeneous resource advantages. It is of great significance to explore the interaction between the local government and social organizations and to clarify the key factors affecting the participation of social organizations in natural hazard emergency responses. With the aim of exploring the relationship between the local government and social organizations, based on evolutionary game theory, the emergency incentive game model and the emergency linkage game model of natural hazard emergency responses were constructed. The evolutionary trajectories of the emergency incentive game system and the emergency linkage game system were described by numerical simulation. Meanwhile, the influence mechanism of government decision parameters on the strategy selection of both game subjects was analyzed. The results show that both governmental incentive strategy and linkage strategy can significantly improve the enthusiasm of social organizations for participating in natural hazard emergency responses. Moreover, they could encourage social organizations to choose a positive participation strategy. Nevertheless, over-reliance on incentives reduces the probability of the local government choosing a positive emergency strategy. In addition, we found that, when both game subjects tend to choose a positive strategy, the strategy selection of the local government drives that of social organizations.

## 1. Introduction

Natural hazards have an impact on both nature and society. The occurrence of natural hazards poses a great threat to social development and public security [1,2]. In recent years, with the superposition and coupling of various risk factors, natural hazards have presented more obvious chain, compound, and derivative characteristics. These characteristics suggest that natural disasters generate a “cascade” of damaging factors, which require the cooperation of multiple departments [3]. In order to effectively deal with natural hazards, implement emergency rescue operations, and reduce the losses of natural hazards, emergency organizations have formed an institutional and spontaneous cooperation mechanism in response to natural hazards in China [4]. Specifically, the external manifestations of the emergency cooperation mechanism in China are cross-organizational, cross-regional, and cross-sectoral [5,6].

In recent years, in order to build a socialized disaster prevention, mitigation, and relief pattern with multiple-party participation, governments around the world have encouraged and guided social organizations to participate in emergency operations in natural hazards with active deployment [7]. Emergency experiences around the world have shown that, with their strong applicability, high operating efficiency, flexible organizational structure, and rapid collection of resources, social organizations have come to play an important role in the governmental emergency response system [8,9]. In the emergency response, social organizations, such as the emergency “linkage partner” and “supplementary force” of governments, have effectively made up for the shortage of government emergency response resources from the practice of emergencies [10].

At present, supporting social organizations in participating in emergency responses has become a common trend among governments globally when dealing with natural hazards. Making full use of the heterogeneous information and external resources of social organizations is helpful for improving the natural hazard emergency response level, a fact that has been recognized by theoretical scholars and government leaders. Therefore, both academic circles and government agencies in China have explored how to improve the emergency response efficiency of social organizations, standardize the participation process of social organizations, and establish a long-term mechanism for social organizations to participate in emergency responses [11,12]. However, it can be known from the emergency practice of the Wenchuan earthquake that occurred in Sichuan Province on 12 May 2008, the Ludian earthquake that occurred in Yunnan Province on 3 August 2014, and the Lushan earthquake that occurred in Sichuan Province on 20 April 2013, there are still some problems that arise when social organizations participate in the emergency response to natural hazards, such as a disorganized response and insufficient guarantee of resources. At the same time, existing research focuses on the participation mode of social organizations and neglects explaining the participation mechanisms of social organizations. Even though some studies have paid attention to the cooperation mechanism between government departments and social organizations, they have not focused on the dynamic characteristics of the cooperation mechanism, to a certain extent. In this context, this research attempted to solve the following three problems:

(1) What is the interaction between the government and the social organizations in natural hazard emergency response and operation?
(2) Can the government’s incentive strategy and linkage strategy improve the enthusiasm and efficiency of the social organization participating in natural hazard emergency operations?
(3) How do these factors influence the implementation effect of government incentive strategy and linkage strategy?

In order to fill this gap in the research, this research built a game model of the local government and social organizations based on evolutionary game theory. By analyzing the possible strategic combination of the local government and social organizations, the dynamic decision-making mechanism of two game subjects was analyzed. On this basis, a numerical simulation was used to describe the evolution model of the game system in which social organizations participate in the emergency response to natural hazards. At the same time, we explored the influence mechanism of strategy parameters on the strategy selection of game subjects and summarized the interactions between the local government and social organizations in emergency cooperation on natural hazards. Furthermore, according to the research results and the practice of natural hazard emergency responses, countermeasures and suggestions are proposed. In general, the theoretical and practical contributions of this article are organized as follows. This study extends the strategy of the government guiding the social organizations to participate in natural hazard emergency response to incentive and linkage. An evolutionary game model to discuss the interaction between the government and the social organizations is established. At the same time, the key factors affecting the strategy selection of the government and the social organizations are identified. This article gives a more direct and accurate description of the strategy selection behavior of the observed game subjects. Furthermore, the article discusses the evolutionary path of the combination of the government and the social organizations to attain a positive strategy through dynamic perspective and numerical simulation analysis. The key factors to improve the efficiency of emergency cooperation and stimulate the initiative of social organizations in emergency response are discussed. In addition, the countermeasures and suggestions proposed by the research provide a decision-making basis for the government’s natural disaster emergency practice.

The framework of this article is as follows: Section 2 focuses on a review of the literature concerned with the participation of social organizations in emergency responses and the application of evolutionary game theory in organizational cooperation; comments on existing research results in related fields are also provided. Section 3 describes the research questions related to the emergency response cooperation between local government and social organizations; the basic hypotheses of this study are presented and explained here. In Section 4 and Section 5, the emergency incentive model and the emergency linkage model for social organizations to participate in natural hazard responses are designed, respectively. Furthermore, the effects of the government’s incentive and linkage strategies are analyzed by numerical simulation, and the influence of different strategy parameters on the strategy selection of the local government and social organizations are discussed. Section 6 presents the discussion, which combines and extends the research results and proposes future research directions. Section 7 summarizes the conclusions; here, we also propose countermeasures and suggestions to improve the level of emergency response cooperation between the local government and social organizations, considering three different perspectives.

## 2. Literature Review

Natural hazards are characterized by suddenness, dynamics, complexity, extensiveness, and uncertainty [13]. Meteorological events, earthquakes, floods, and typhoons are the main types of natural hazards [9,14,15]. Therefore, there is an urgent need to take corresponding measures in the emergency management of natural hazards. According to the practice of natural hazard emergency response, in order to meet the needs of natural hazard emergency and disposal, different types and levels of emergency response organizations have formed institutional or spontaneous multi-organization cooperation mechanisms [16]. From the practice of natural disaster emergency around the world, the cooperation of emergency response organizations during natural hazards mainly includes the vertical cooperation among government departments at different levels, horizontal cooperation among government departments at the same level, and cross-border cooperation between government departments and social organizations or the military [17,18,19]. As an important part of emergency cooperation during natural hazards, the cooperative relationship between the government and social organizations is the key research object of this paper.

### 2.1. Participation of Social Organizations in Emergency Responses

Social organizations are characterized by public welfare, professionalism, and voluntariness [20,21]. At the same time, social organizations have shown remarkable advantages in participating in emergency actions, such as strong mobility, low communication costs, outstanding organization ability, diversified resources, and strong matching applicability [22]. Therefore, social organizations can make up for a government’s shortcomings in emergency operations [23]. The U.S. government attributes great importance to the coordination and cooperation between interstate governments and relevant functional departments. At present, following the formulation of the Emergency Management Assistance Compact, the U.S. government encourages nonprofit and public organizations to participate in emergency responses and rescue [24]. The Chinese government also pays great attention to the emergency rescue work during natural hazards. The relevant departments have formulated and revised several editions of the Regulations on Natural Hazard Relief and the National Emergency Plan for Natural Hazard Relief and have deployed specific tasks to guide social emergency forces to participate in disaster relief work [25].

In the existing research in China, the scenes of social organizations participating in emergency operations are mostly of natural hazards and public health emergencies. Most of the related research results are based on actual cases [26,27]. For natural hazards, relevant scholars have summarized and combed the practical experience of social organizations participating in emergency rescue operations, such as response to earthquake disasters, geological disasters, floods, and meteorological disasters. In public health emergencies, the participation of social organizations is mostly carried out in the form of voluntary services, such as stationing at the checkpoint and checking traffic information.

Previous studies have shown that, at different emergency stages, social organizations take on different responsibilities, functions, and tasks when participating in emergency operations. In the stage of emergency preparedness and prevention, social organizations play a positive role in emergency science popularization, emergency publicity and education [28], emergency public welfare training, and participation in joint drills. In the stage of emergency response and disposal, social organizations mainly participate in emergency functions such as emergency rescue, emergency supplies, emergency support, and emergency communication. They also assist with tasks of medical assistance, evacuation, and temporary on-site control. In addition, social organizations have participated in emergency operations such as psychological counseling, material donations, and disaster site reconstruction in the stages of emergency recovery and aftermath. At the same time, different types of social organizations play different roles in emergency management.

In relevant policies, the Chinese government divides social organizations into four types—namely, professional social organizations, hub social organizations, foundations, and industry associations. Correspondingly, different types of social organizations are responsible for professional services, docking information, financial and material support, industry management, and other tasks.

Furthermore, in order to explore the path of social organizations participating in emergency response operations and the cooperation mode between government departments and social organizations, some scholars have introduced the social network analysis method to reconstruct an emergency cooperation network in China. By analyzing the overall structure and core attributes of the emergency cooperation network, the types and levels of social organizations participating in emergency responses are condensed. The corresponding relationships between social organizations and emergency functions are here summarized, and the role of social organizations in emergency operations are identified. Chen et al., based on the Ya’an earthquake, reconstructed the institutionalized emergency correlation network before and the dynamic emergency response network after the earthquake. Then, they revealed the main role of social organizations in emergency responses [29]. Zhang et al. analyzed the cross-sectoral interaction between public organizations, private organizations, and nonprofit organizations by constructing a complex adaptive network based on the Lushan earthquake [30]. Lian et al. reconstructed the cooperation network of emergency support based on the extensive participation of social organizations from China’s practice in responding to COVID-19. Their research study explored the growth in the emergency cooperation network [31]. Du et al. analyzed the dynamic development of the emergency response network by taking the chemical plant explosion accident of Xiangshui as an example. They concluded that nonprofit organizations are on the outer edge of the emergency response network and asserted that the cooperation between public sectors and nonprofit organizations requires further improvement [32]. Lu et al. reconstructed a disaster relief network of nonprofit organizations acting during small- and medium-sized natural hazards by taking One Foundation as an example. In their article, the network structure and operation status were analyzed and the sustainable mode of network expansion was condensed [33].

To sum up, the existing studies focus more on the overall model of the cooperative relationship between government departments and social organizations, or explore the development of the cooperative relationship between the two. However, to some extent, the existing research ignores the ways in which government decision-making mechanisms influence and differ from the initiative and enthusiasm of social organizations’ participation in emergency response operations. An analysis of how social organizations effectively support governments in providing emergency responses is also lacking. At the same time, the relevant research results are mostly static descriptions of the cooperative relationship between governments and social organizations. Research results from the perspective of dynamic evolution are less commonly found in the literature.

### 2.2. Application of Evolutionary Game Theory in Organization Cooperation

In emergency responses and operations during natural hazards, the government and social organizations form a certain degree of cooperation and exchange. At the same time, in the selection of an emergency strategy, the government and social organizations influence one another. In order to explore the cooperative behavior between the government and social organizations in emergency responses, this paper introduces evolutionary game theory to characterize the relationship between them [34]. Thus far, evolutionary game theory has been applied to describe cooperation patterns and to analyze cooperative relations among various organizations [35].

In the field of organizational cooperation, evolutionary game theory has been widely applied to practical problems of Chinese organization cooperation, such as industry–university–research cooperation [36], market supervision cooperation [37], environmental governance cooperation [38], and sustainable humanitarian supply chain [39]. Of course, it also includes the topic of emergency management cooperation [40,41,42]. By summarizing the competition and cooperative relationship between organizations, scholars have reconstructed the corresponding evolutionary game model and have discussed the strategy selection mode adopted by each subject in the process of organizational cooperation.

From the perspective of organizational cooperation level, previous research studies in China have mainly focused on inter-governmental or inter-departmental cooperation, cooperation between the government and enterprises, cooperation between government and nongovernmental organizations, cooperation among scientific research institutions, and cooperation between transnational governments and other inter-organizational relations. The formation and reciprocal influence mechanisms of inter-organizational cooperation have been explored. At the same time, in the study of inter-governmental or inter-departmental cooperation, there are also other research results relative to cross-regional and cross-departmental cooperation and horizontal and vertical cooperation [43].

It is particularly important for this study to deeply consider the cooperative relationship and mutual influence between governments and social organizations in the emergency response to natural hazards. At the present stage, relevant scholars have made efforts in exploring the cooperative relationship between social organizations and other organizations during emergencies, as shown in Table 1.

Chen et al. established an evolutionary game model between international nongovernment organizations and local nongovernment organizations during disasters, which described the coordination relationship between international nongovernment organizations and local nongovernment organizations in the humanitarian supply chain. The corresponding suggestions to improve the relief efficiency and sustainability of the humanitarian supply chain were put forward [44]. Xu et al. discussed the game and cooperation among governments, enterprises, and public departments in public health emergencies and proposed countermeasures and suggestions to promote three-agent cooperation in dealing with public health emergencies [45]. From the perspective of disaster emergency mobilization, Du and Qian analyzed the interaction mechanism and influencing factors among governments, government-owned NPOs (GONPOs), and grassroots NPOs (GRNPOs) in disaster emergency relief [46]. Hou and Lv described the embedded cooperation between government and nonprofit organizations in geological disaster emergency management based on evolutionary game theory. The influencing factors of the cooperative relationship between governments and nonprofit organizations were analyzed [47].

Therefore, evolutionary game theory can clearly and intuitively analyze the cooperative and interactive relationship between government and social organizations in the emergency response to natural hazards. Therefore, in this article, we built a game model for social organizations to participate in the emergency response to natural hazards. In order to further describe the practical effect of strategy implementation as operated by each game subject, the influence of different strategies on the evolution of the game system is discussed visually through simulation analysis. At the same time, this study also focused on the evolution of the interaction between government and social organizations in emergency decision making. Furthermore, this study aimed to explore the government’s own evolution behavior and its influence on the strategy selection performed by social organizations.

## 3. Research Problem Description and Basic Hypotheses

### 3.1. Proposal of Research Questions

Both the local government and social organizations are important components of emergency practice during natural hazards. As the main subject of command and the core force in the response to natural hazards, the local government is responsible for emergency command, emergency rescue, emergency support, and other emergency functions. Social organizations, mainly composed of public welfare associations and voluntary organizations, are auxiliary forces that help deal with natural hazards. In the emergency response to natural hazards, in order to encourage and take advantage of the supporting role that social organizations play in the government and its functional departments’ plans, the local government can implement incentive and linkage strategies aimed at social organizations. Incentive and linkage strategies jointly construct the interaction framework of the local government and social organizations. Among them, incentive strategies mainly include compensation, economic, reputation, and other incentives. Meanwhile, linkage strategies can be divided into the linkage of emergency preparedness and the linkage of emergency response.

According to the practice of natural hazard emergency response, the key to improving the performance of emergency responses is to enhance the cooperation efficiency and coordination level between the local government and social organizations. Therefore, in this paper, we built the emergency incentive model and the emergency linkage model. The interaction between the local government and social organizations in natural hazard emergency responses and operations during natural hazards was thoroughly analyzed. The influence mechanism of the local government’s incentive and linkage strategies on social organizations’ participation in the emergency response to natural hazards can be further explored; therefore, this paper provides theoretical support for summarizing practical suggestions to improve the level of emergency cooperation between the local government and social organizations.

### 3.2. Research Hypotheses

**Figure 1:**
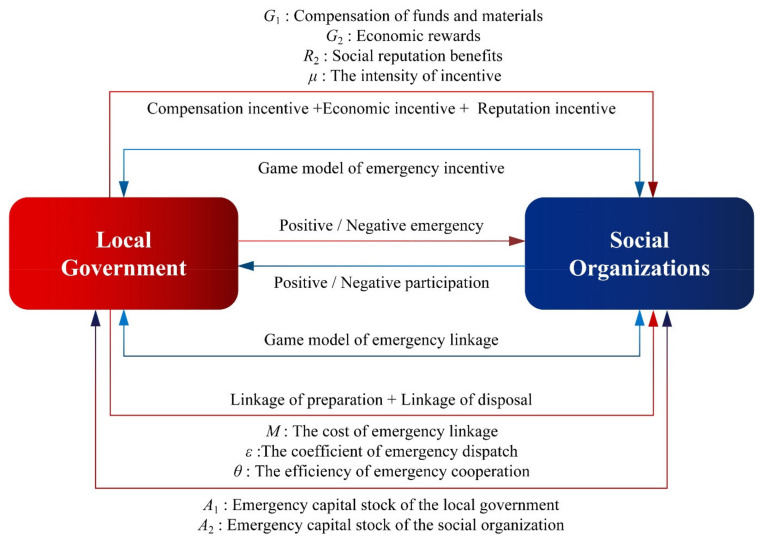
The game relationship between the local government and social organizations.

In this article, we built an evolutionary game model focusing on the local government and social organizations in their emergency response to natural hazards. In order to clarify the specific expression of the local government’s emergency strategy and social organizations’ participation strategy in the emergency incentive game model and the emergency linkage game model, the following hypotheses are proposed. The game relationship between the local government and social organizations is shown in Figure 1.

**Hypothesis** **H1.** *In the emergency incentive model and the emergency linkage model, both the local government and social organizations are bounded rationally. The local government and social organizations choose strategies based on specific transmission mechanisms, rather than rational choice. Moreover, there is information asymmetry between them. In the process of evolution, the local government and social organizations play random matches and repeated games.*

**Hypothesis** **H2.** *The local government, as the main subject of responsibility for the emergency response to natural hazards, is required to be responsible for emergency command and unified coordination. On the one hand, the local government can choose a positive emergency strategy to minimize the losses caused by natural hazards and to safeguard people’s lives and property. On the other hand, because the costs of emergency operations, incentive expenditure, and emergency linkage are relatively high, or, conversely, the income of emergency operations is low, this reduces the local government’s initiative in choosing a positive emergency strategy. Therefore, the local government chooses negative emergency strategies.*

**Hypothesis** **H3.** *After the occurrence of natural hazards, social organizations emerge as beneficial supplements to the government’s emergency response systems. Social organizations cover an important role in supporting the government in implementing emergency rescue and guaranteeing emergency operations. In the practice of natural hazard emergency responses, social organizations may choose a positive participation strategy due to the high perception of losses and various incentive benefits. On the contrary, it is also possible for them to choose a negative participation strategy because of the high costs and low incentive benefits for emergency operations.*

**Hypothesis** **H4.** *In the emergency incentive game model, the probability that the local government chooses a positive and a negative emergency strategy is x and 1 − x (0 ≤ x ≤ 1), respectively. The probability that social organizations choose a positive and a negative participation strategy is y and 1 − y (0 ≤ y ≤ 1), respectively. Similarly, in the emergency linkage game model, the probability that the local government chooses a positive and a negative emergency strategy is p and 1 − p (0 ≤ p ≤ 1), respectively. The probability that social organizations choose a positive and negative participation strategy is q and 1 − q (0 ≤ q ≤ 1), respectively.*

## 4. Construction and Simulation Analysis of the Emergency Incentive Game Model

### 4.1. Parameter Settings of the Emergency Incentive Game Model

In this paper, we built an emergency incentive game model with compensation, economic, and reputation incentives. The game subjects are the local government and social organizations. In order to clarify the dynamic selection mechanism between the local government and social organizations in the emergency incentive game, the following parameter settings are proposed. Table 2 shows the definition and ranges of each parameter in the emergency incentive game model.

Setting 1. The local government pays for the organization, equipment, manpower, and other emergency costs *C*_1_ linked to emergency responses and operations during natural hazards. Meanwhile, emergency benefits *T*_1_ and social reputation benefits *R*_1_ are obtained. When social organizations participate in natural hazard emergency responses, they pay for the emergency organization and coordination costs *C*_2_. At the same time, this brings additional emergency benefits *T*_2_ to the local government. *L*_1_ is the sum of the local government’s perception of social development losses, direct property losses, and human casualty losses caused by natural and derivative disasters. *L*_2_ represents the sum of the social organizations’ perception of the possible adverse effects of natural and derivative disasters.

Setting 2. In the emergency incentive game model, this research study describes three aspects of incentive measures: Compensation, rewards, and reputation incentives. First, when social organizations participate in natural hazard emergency responses and operations, the local government provides social organizations compensation *G*_1_ for the wastage of funds, materials, and equipment. Second, when social organizations choose the strategy of positive emergency participation, the local government provides social organizations a certain economic reward *G*_1_. Third, the local government grants commendations to social organizations participating in emergency operations during the publicity and action summary activities of a natural hazard emergency. Then, social organizations gain trust benefits and social reputation benefits *R*_2_. At the same time, the local government pays for some incentive costs *W* for implementing various incentive measures: *W* = *G*_1_ + *G*_2_.

Setting 3. In order to measure the level of emergency response and operations implemented by the local government, this paper introduces the local government’s emergency intensity *α*. A higher value of the local government’s emergency intensity indicates that the emergency costs paid by the local government are greater and that the emergency benefits and reputation benefits obtained are higher. When the local government chooses a positive emergency strategy, *α* = 1. When the local government chooses a negative emergency strategy, the emergency costs paid by the local government are *αC*_1_, and the emergency benefits and reputation benefits obtained are *αT*_1_ and *αR*_1_, respectively.

Setting 4. In order to describe social organizations’ initiative to participate in emergency operations, we defined *β* as the social organizations’ participation intensity. When the value of participation intensity *β* is higher, the emergency costs paid by social organizations rise and the compensation income and reputation income obtained by social organizations and the emergency benefits obtained by the local government from social organizations increase. When social organizations choose a positive participation strategy, *β* = 1. Furthermore, when social organizations choose a negative participation strategy, the emergency costs paid by social organizations are *βC*_2_, the compensation benefits and reputation benefits obtained are *βG*_1_ and *βR*_2_, respectively, and the extra emergency benefits obtained by the local government are *βT*_2_.

Setting 5. In order to depict the incentive level adopted by the local government with respect to social organizations, we defined *μ* as the local government’s incentive intensity. A high value of incentive intensity *μ* means that the local government pays for more incentive costs and that the social organizations obtain more compensation incentives, reward incentives, and reputation incentives. When the local government chooses a positive emergency strategy, *μ* = 1. Therefore, the incentive costs of the local government under the negative emergency strategy refer to *μM*, and the compensation, rewards, and reputation incentives obtained by social organizations are *μG*_1_, *μG*_1_, and *μR*_1_, respectively.

### 4.2. Payoff Matrix of the Emergency Incentive Game Model

Based on the emergency incentive game model of the local government and social organizations, the benefits of the local government and social organizations under different game strategy combinations were solved. The payoff matrix of the emergency incentive game model was obtained after calculations, as shown in Table 3.

### 4.3. Replication Dynamic Equation of the Emergency Incentive Game Model

#### 4.3.1. Replication Dynamic Equation of the Local Government

According to the payoff matrix, the expected benefit of the local government choosing the positive emergency strategy *U*_11_ is as follows:

(1) $$U_{11}=y\left(-C_{1}-L_{1}-W+T_{1}+R_{1}+T_{2}\right)+\left(1-y\right)\left(-C_{1}-L_{1}-W+T_{1}+R_{1}+\beta T_{2}\right)$$

Then, the expected benefit of the local government choosing a negative emergency strategy *U*_12_ is as follows:

(2) $$U_{12}=y\left(-\alpha C_{1}-L_{1}-\mu W+\alpha T_{1}+\alpha R_{1}+\mu T_{2}\right)+\left(1-y\right)\left(-\alpha C_{1}-L_{1}-\mu W+\alpha T_{1}+\alpha R_{1}+\mu \beta T_{2}\right)$$

Therefore, it can be known that the replication dynamic equation of the local government’s emergency strategy is:

(3) $$F\left(x\right)=\frac{dx}{dt}=x\left(1-x\right)\left(U_{11}-U_{12}\right)=x\left(1-x\right)\left[-\left(1-\alpha \right)\left(C_{1}-T_{1}-R_{1}\right)-\left(1-\mu \right)\left(W-\beta T_{2}\right)+y\left(1-\beta \right)\left(1-\mu \right)T_{2}\right]$$

#### 4.3.2. Replication Dynamic Equation of Social Organizations

Likewise, the expected benefit of social organizations choosing a positive participation strategy *U*_21_ is as follows:

(4) $$U_{21}=x\left(-C_{2}-L_{2}+G_{1}+G_{2}+R_{2}\right)+\left(1-x\right)\left(-C_{2}-L_{2}+\mu G_{1}+\mu G_{2}+\mu R_{2}\right)$$

The expected benefit of social organizations choosing a negative participation strategy *U*_21_ is as follows:

(5) $$U_{22}=x\left(-\beta C_{2}-L_{2}+\beta G_{1}+\beta R_{2}\right)+\left(1-x\right)\left(-\beta C_{2}-L_{2}+\mu \beta G_{1}+\mu \beta R_{2}\right)$$

Then, the replication dynamic equation of social organizations’ participation strategies can be obtained as follows:

(6) $$G\left(y\right)=\frac{dy}{dt}=y\left(1-y\right)\left(U_{21}-U_{22}\right)=y\left(1-y\right)\left\{-\left(1-\beta \right)\left(C_{2}-\mu G_{1}-\mu R_{2}\right)+\mu G_{2}+x\left(1-\mu \right)\left[\left(1-\beta \right)\left(G_{1}+R_{2}\right)+G_{2}\right]\right\}$$

### 4.4. Equilibrium Point of the Emergency Incentive Game Model

The replication dynamic equation can reflect the behavioral interaction of both game subjects and the direction and speed of the strategic game. When the replication dynamic equation of both game subjects is 0, the replication dynamic system forms an equilibrium state. Therefore, setting *F*(*x*) = 0 and *G*(*y*) = 0, five equilibrium points of the replication dynamical system of the emergency incentive game model can be obtained, which are *O* (0, 0), *A* (1, 0), *B* (1, 1), *C* (0, 1), and *D* (x*, y*), respectively, including $x^{*}=\frac{\left(1-\beta \right)\left(C_{2}-\mu G_{1}-\mu R_{2}\right)-\mu G_{2}}{\left(1-\mu \right)\left[\left(1-\beta \right)\left(G_{1}+R_{2}\right)+G_{2}\right]}$, $y^{*}=\frac{\left(1-\alpha \right)\left(C_{1}-T_{1}-R_{1}\right)+\left(1-\mu \right)\left(W-\beta T_{2}\right)}{\left(1-\beta \right)\left(1-\mu \right)T_{2}}$.

**Figure 2:**
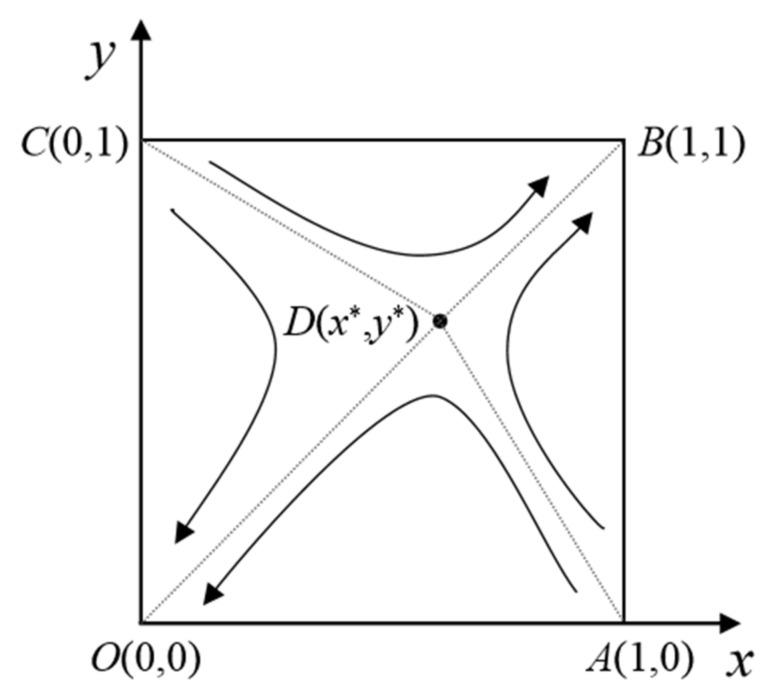
Evolutionary phase diagram of the emergency incentive system.

The influence of the initial state on the evolution trend of the system is analyzed according to the stability theory of differential equations. The evolutionary phase diagram of the emergent incentive game system is shown in Figure 2. When *x* > x*, the equations $G^{\prime }\left(y\right)\left|{}_{y=1}\right.<0$ and $G^{\prime }\left(y\right)\left|{}_{y=0}\right.>0$ are true. At this point, *y* = 1 (positive participation) is the steady state of the game system. On the contrary, when *x* < x*, both $G^{\prime }\left(y\right)\left|{}_{y=1}\right.>0$ and $G^{\prime }\left(y\right)\left|{}_{y=0}\right.<0$ are true. Therefore, *y* = 0 (negative participation) is the steady state of the game system. Similarly, when *y* > y*, *x* = 1 (positive emergency) is the steady state of the game system. When *y* < y*, *x* = 0 (negative emergency) is the steady state of the game system.

### 4.5. Simulation Analysis of the Emergency Incentive Game Model

In order to intuitively analyze the influence of the local government’s different incentive measures on the main subject’s strategy selection in the emergency incentive game system, a numerical simulation analysis was carried out. In this paper, the initial values of each parameter in the model were determined by combining the statistical data from the *China Statistical Yearbook* and Delphi analysis based on expert scoring. Let us assume that *α* = 0.5, *β* = 0.5, *μ* = 0.5, *C*_1_ = 15, *C*_2_ = 15, *R*_1_ = 3, *R*_2_ = 5, *T*_1_ = 9, *T*_2_ = 18, *G*_1_ = 5, and *G*_2_ = 5. Meanwhile, the initial probabilities of the strategy selection of the local government and social organizations in the game system were set as 0.5.

#### 4.5.1. The Effect of *β* on the Evolution of the Emergency Incentive Game System

**Figure 3:**
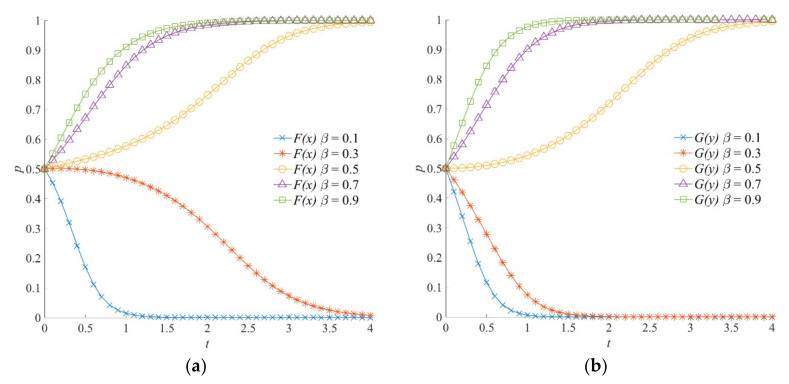
Evolution trajectory of the emergency incentive game system under different values of social organizations’ participation intensity. (**a**) The influence of *β* on the evolution of the local government’s strategy. (**b**) The influence of *β* on the evolution of social organizations’ strategies.

The evolutionary trajectories of the emergency incentive game system, considering different values of social organizations’ participation intensity, are shown in Figure 3. When *β* = 0.1 or *β* = 0.3, the emergency incentive game system converges to (0, 0). When *β* is 0.5, 0.7, and 0.9, local government and social organizations evolve into positive strategies and form a stable strategy combination (1, 1). According to the emergency incentive game model, with the increase in participation intensity *β*, the perception of emergency benefits gained by the local government due to the participation of social organizations in emergency operations increases. At the same time, social organizations obtain more compensation and social reputation benefits from the local government.

Therefore, consistent with the trend shown in Figure 3a,b, when the social organizations’ participation intensity is greater, the local government and social organizations have a higher probability of choosing positive emergency strategies. Meanwhile, by comparing Figure 3a,b, when *β* is 0.1 and 0.3, the convergence speed of social organizations toward the negative participation strategy is faster than that of the local government toward the negative emergency strategy. On the contrary, when (1, 1) is the stable strategy of the emergency incentive game system, the local government shows a faster speed in converging to the positive emergency strategy. This indicates that the local government is more sensitive to an increase in participation intensity and that social organizations are more sensitive to a decrease in participation intensity. The more sensitive the game subject is, the faster the strategy changes.

#### 4.5.2. The Effect of *μ* on the Evolution of the Emergency Incentive Game System

**Figure 4:**
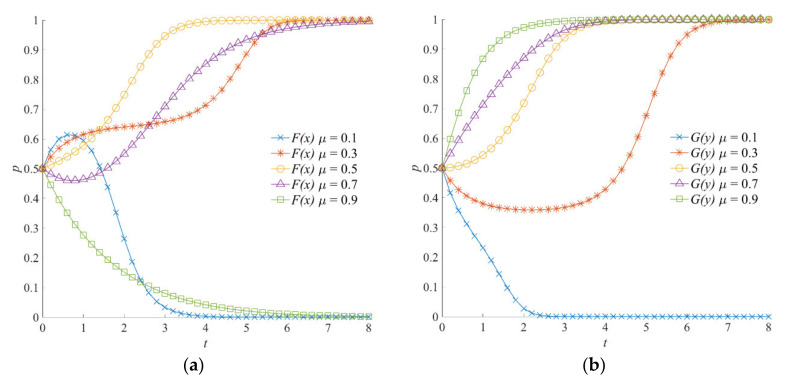
Evolution trajectory of the emergency incentive game system under different values of the local government’s incentive intensity. (**a**) The influence of *μ* on the evolution of the local government’s strategy. (**b**) The influence of *μ* on the evolution of social organizations’ strategies.

The simulation results of the emergency incentive game system when the incentive intensity of the local government *μ* is 0.1, 0.3, 0.5, 0.7, and 0.9 are shown in Figure 4. As shown in Figure 4a, when *μ* = 0.1 or *μ* = 0.9, the negative emergency strategy is the evolutionarily stable strategy of the local government. In other words, with the increase in incentive intensity *μ*, the strategy evolution of the local government goes from “negative” to “positive” and then to “negative”.

When the incentive intensity is low, the local government shows a weak incentive effect on social organizations. At this time, the emergency benefits obtained from social organizations’ participation in an emergency are low, so the local government chooses a negative emergency strategy. Meanwhile, although the increase in incentive intensity may temporarily encourage the local government to choose a positive emergency strategy, the local government reduces the initiative of implementing the positive emergency strategy due to having to cover more incentive costs, given the high value of incentive intensity. This shows that, for the emergency incentive game system, whether the incentive intensity is too high or too low is not conducive to the formation of a positive strategy combination. On the contrary, an appropriate incentive intensity is required to improve the level of emergency coordination between the local government and social organizations. Figure 4b shows that, with the increase in incentive intensity, social organizations have higher perception of compensation, rewards, and reputation benefits from the local government and the public. Therefore, social organizations show a shift from a negative participation strategy to a positive participation strategy. At the same time, the convergence rate of active participation strategy of social organizations is faster at a high level of incentive intensity.

#### 4.5.3. The Effect of Incentives on the Evolution of Social Organizations’ Strategies

##### The Compensation Incentive

**Figure 5:**
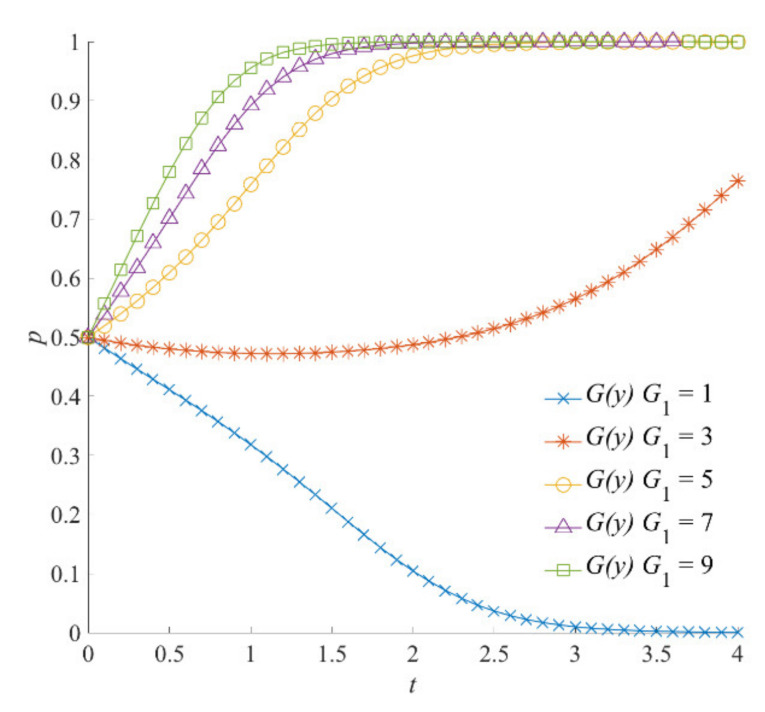
The compensation *G*_1_ on the evolution of social organizations’ strategies.

Under different compensation levels dispensed by the local government, the evolution results of the social organizations’ participation strategies are shown in Figure 5. When *G*_1_ = 1, the social organizations involved in natural hazard emergency responses need to pay large costs for emergency operations and fail to obtain sufficient fund compensation and material compensation. In this case, the negative participation strategy is the evolutionarily stable strategy of social organizations. When *G*_1_ = 3, the perception of the compensation benefits of social organizations increases and the enthusiasm of social organizations for choosing a positive participation strategy is also enhanced. As the evolution time increases, it converges to *y* = 1. Furthermore, when *G*_1_ is 5, 7, or 9, social organizations show greater initiative for participating in emergency operations. The evolutionary results of social organizations’ strategy selection all indicate positive participation strategies. Noticeably, when *G*_1_ = 9, the evolution time of social organizations toward a positive participation strategy is the shortest and the fastest.

##### The Reward Incentive

**Figure 6:**
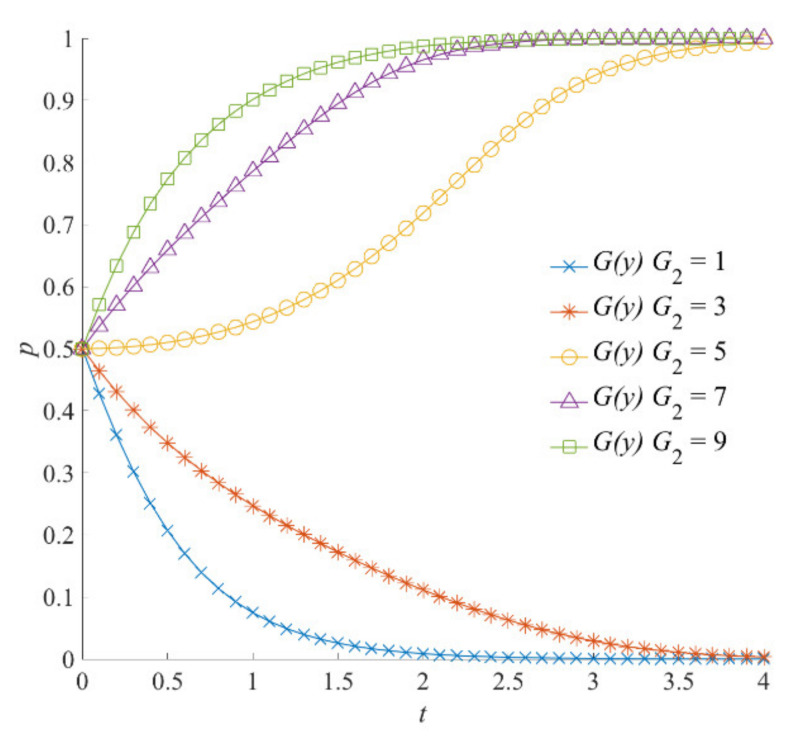
The rewards *G*_2_ on the evolution of social organizations’ strategies.

Under different reward levels dispensed by the local government, the evolutionary trajectories of the social organizations’ participation strategies are shown in Figure 6. With the increase in *G*_2_, the participation strategy of social organizations shows a shift from negative to positive. When social organizations choose a positive participation strategy, they can obtain certain economic rewards from the local government. Therefore, the effect of the local government’s incentive *G*_2_ on the social organizations’ strategy selection is different from that of *G*_1_. On the contrary, when *G*_1_ is 1 and 3, social organizations show a shift from a negative to a positive strategy; however, the transformation path of *G*_2_ is between the values of 3 and 5. On the contrary, the evolution of social organizations’ participation strategy to stable strategy in *G*_1_ is faster than that in *G*_2_. In conclusion, this shows that the promotion of *G*_2_ has little influence on the change in social organizations’ strategies.

##### The Reputation Incentive

**Figure 7:**
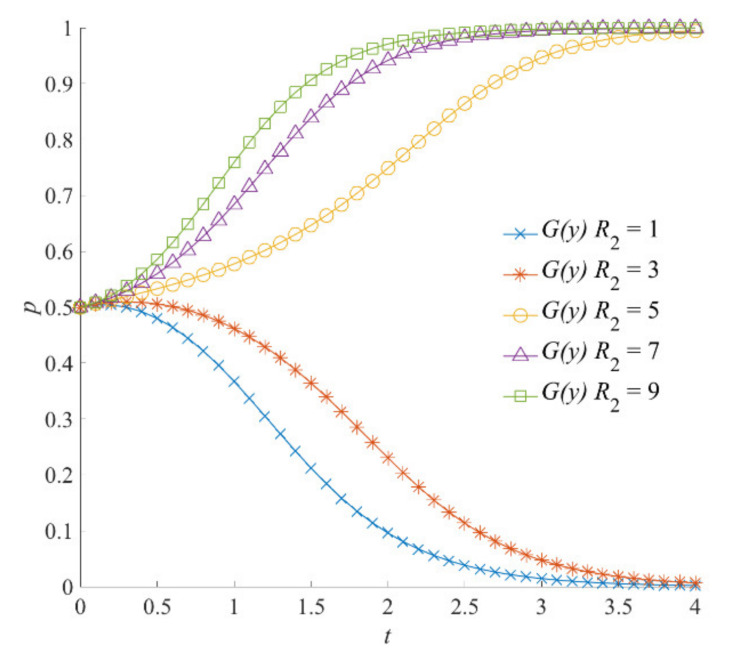
The reputation benefits *R*_2_ on the evolution of social organizations’ strategies.

The simulation results of social organizations’ strategies, when the social reputation benefits obtained by social organizations *R*_2_ are 1, 3, 5, 7, and 9, are shown in Figure 7. When *R*_2_ is 1 and 3, social organizations are more likely to choose a negative participation strategy. With the increase in simulation time, the participation strategy of social organizations converges to *y* = 1. When *R*_2_ is set to 5, 7, and 9, social organizations tend to choose a positive participation strategy because they obtain higher social reputation benefits due to the local government’s recognition and publicity. However, by comparing Figure 5, Figure 6 and Figure 7, it can be seen that the influence mechanism of *R*_2_’s promotion on social organizations’ strategy selection is different from that of *G*_1_ and *G*_2_. The influence effect of social reputation benefits on social organizations’ strategy selection is lagging behind. At the same time, in the early stage of evolution, there is a delay in the strategy evolution of social organizations—a temporary stall. This shows that the compensation and reward given by the local government have a more direct impact on the enthusiasm of social organizations to participate in the emergency response to natural hazards.

## 5. Construction and Simulation Analysis of the Emergency Linkage Game Model

### 5.1. Parameter Settings of the Emergency Linkage Game Model

In this paper, we built an emergency linkage game model based on the linkage between the stages of preparation and disposal of a natural hazard emergency. In order to analyze the strategy selection modes of the local government and social organizations under different strategy combinations, the following parameter settings are proposed. Table 4 shows the definitions and ranges of the parameters involved in the emergency linkage game model.

Setting 1. After a natural hazard, the local government should pay for the costs of emergency operations *C*_1_ due to the implementation of the emergency response. At the same time, the local government obtains a certain benefit *T*_1_ from emergency operations. Social organizations pay for the cost of equipment, materials, and manpower *C*_2_ in order to cooperate and support the local government’s emergency response. The local government gains additional emergency linkage benefits *T*_2_ from the support and cooperation of social organizations. When a natural hazard occurs, the perceptions of the local government and social organizations of the various losses caused by natural and derivative disasters are *L*_1_ and *L*_2_, respectively.

Setting 2. In this study, we designed the local government’s emergency linkage strategy based on two aspects: preparedness linkage and response linkage. On the one hand, the local government and social organizations implement the linkage of emergency preparedness in the aspects of emergency plan preparation, emergency rescue drills, and emergency dispatching platform application. At this point, the local government and social organizations form emergency capital stock *A*_1_ and *A*_2_, respectively, based on institutional norms and cooperative trust. When the emergency capital stock is greater, the emergency costs paid by the local government and social organizations in the practice of natural hazard emergency responses decrease. On the other hand, the local government and social organizations form emergency linkage tasks based on disaster situation, disaster type, and emergency demand during the implementation of emergency responses and disposal. We defined the coefficient of emergency dispatching *ε* and the degree of emergency cooperation *θ* to describe the practice of the emergency response linkage between the local government and social organizations. In addition, in the linkage of emergency preparation and emergency disposal, the local government needs to pay for the emergency linkage costs *M*, which include drills, plans, platform construction, command, and dispatching.

Setting 3. In the emergency linkage game model, the emergency intensity of the local government *α* is introduced to describe the level of the local government’s emergency operations. At the same time, the participation intensity of social organizations *β* is retained to measure the initiative of social organizations to participate in emergency responses. When the local government chooses a negative emergency strategy, the emergency operation costs and the emergency linkage costs of the local government are *αC*_1_ and *αM*, respectively. The emergency benefits obtained by the local government for implementing emergency responses are *αT*_1_. Meanwhile, the emergency capital stock available to the local government and social organizations are *αA*_1_ and *αA*_2_, respectively. Similarly, when social organizations choose a negative participation strategy, the emergency linkage benefits available to the local government are *βT*_2_. The emergency capital stock available to local government and social organizations are *βA*_1_ and *βA*_2_, respectively.

Setting 4. In order to represent the local government’s emergency command level in the process of natural hazard emergency responses, we defined the coefficient of emergency dispatching *ε*. When the coefficient of emergency dispatching *ε* is high, the local government obtains more emergency linkage benefits. Therefore, in the emergency linkage game model, the emergency benefits obtained by the local government due to its own emergency operations and the participation of social organizations are *εT*_1_ and *εT*_2_, respectively.

Setting 5. In order to measure the implementation effect of natural hazard emergency responses, the degree of emergency cooperation *θ* was defined. When the local government and social organizations choose positive emergency strategies, the matching degree of the local government and social organizations in emergency rescue implementation, equipment, and material coordination is high, given a high value of *θ*. At the same time, with the increase in the degree of emergency cooperation *θ*, the perception of natural hazard losses of the local government and social organizations decreases to (1 − *θ*) × *L*_1_, and (1 − *θ*) × *L*_2_, respectively.

### 5.2. Payoff Matrix of the Emergency Linkage Game Model

Combined with the game scenario in the emergency linkage model between the local government and social organizations proposed in this study, the corresponding payoff matrix of the local government and social organizations under different game strategy combinations was designed, as shown in Table 5.

### 5.3. Replication Dynamic Equation of the Emergency Linkage Game Model

#### 5.3.1. Replication Dynamic Equation of the Local Government

According to the payoff matrix, the expected benefit of the local government choosing a positive emergency strategy *E*_11_ is as follows:

(7) $$E_{11}=q\left[-C_{1}+A_{1}-\left(1-\theta \right)L_{1}-M+\varepsilon T_{1}+\varepsilon T_{2}\right]+\left(1-q\right)\left(-C_{1}+\beta A_{1}-L_{1}-M+\varepsilon T_{1}+\beta \varepsilon T_{2}\right)$$

Then, the expected benefit of the local government choosing a negative emergency strategy *E*_12_ is as follows:

(8) $$E_{12}=q\left(-\alpha C_{1}+\alpha A_{1}-L_{1}-\alpha M+\alpha \varepsilon T_{1}+\alpha \varepsilon T_{2}\right)+\left(1-q\right)\left(-\alpha C_{1}+\alpha \beta A_{1}-L_{1}-\alpha M+\alpha \varepsilon T_{1}+\alpha \beta \varepsilon T_{2}\right)$$

The replication dynamic equation of the local government’s emergency strategy can be obtained:

(9) $$H\left(p\right)=\frac{dp}{dt}=p\left(1-p\right)\left(E_{11}-E_{12}\right)=p\left(1-p\right)\left[-\left(1-\alpha \right)\left(C_{1}+M-\varepsilon T_{1}-\beta A_{1}-\beta \varepsilon T_{2}\right)+q\theta L_{1}+q\left(1-\alpha \right)\left(1-\beta \right)\left(A_{1}+\varepsilon T_{2}\right)\right]$$

#### 5.3.2. Replication Dynamic Equation of Social Organizations

Similarly, the expected benefit of social organizations choosing a positive participation strategy *E*_11_ is as follows:

(10) $$E_{21}=p\left[-C_{2}+A_{2}-\left(1-\theta \right)L_{2}\right]+\left(1-p\right)\left(-C_{2}+\alpha A_{2}-L_{2}\right)$$

Then, the expected benefit of social organizations choosing a negative participation strategy *E*_12_ is as follows:

(11) $$E_{22}=p\left(-\beta C_{2}+\beta A_{2}-L_{2}\right)+\left(1-p\right)\left(-\beta C_{2}+\alpha \beta A_{2}-L_{2}\right)$$

The replication dynamic equation of social organizations’ participation strategies can be obtained as follows:

(12) $$K\left(q\right)=\frac{dq}{dt}=q\left(1-q\right)\left(E_{21}-E_{22}\right)=q\left(1-q\right)\left[-\left(1-\beta \right)\left(C_{2}-\alpha A_{2}\right)+p\theta L_{2}+p\left(1-\alpha \right)\left(1-\beta \right)A_{2}\right]$$

### 5.4. Equilibrium Point of the Emergency Linkage Game Model

In the same way, setting *H*(*p*) = 0 and *K*(*q*) = 0, five equilibrium points of the replication dynamical system of the emergency linkage game model can be obtained, which are *O*’ (0, 0), *A*’ (1, 0), *B*’ (1, 1), *C*’ (0, 1) and *D*’ (x*, y*), respectively, including $p^{*}=\frac{\left(1-\beta \right)\left(C_{2}-\alpha A_{2}\right)}{\theta L_{2}+\left(1-\alpha \right)\left(1-\beta \right)A_{2}}$, $q^{*}=\frac{\left(1-\alpha \right)\left(C_{1}+M-\varepsilon T_{1}-\beta A_{1}-\beta \varepsilon T_{2}\right)}{\theta L_{1}+\left(1-\alpha \right)\left(1-\beta \right)\left(A_{1}+\varepsilon T_{2}\right)}$.

**Figure 8:**
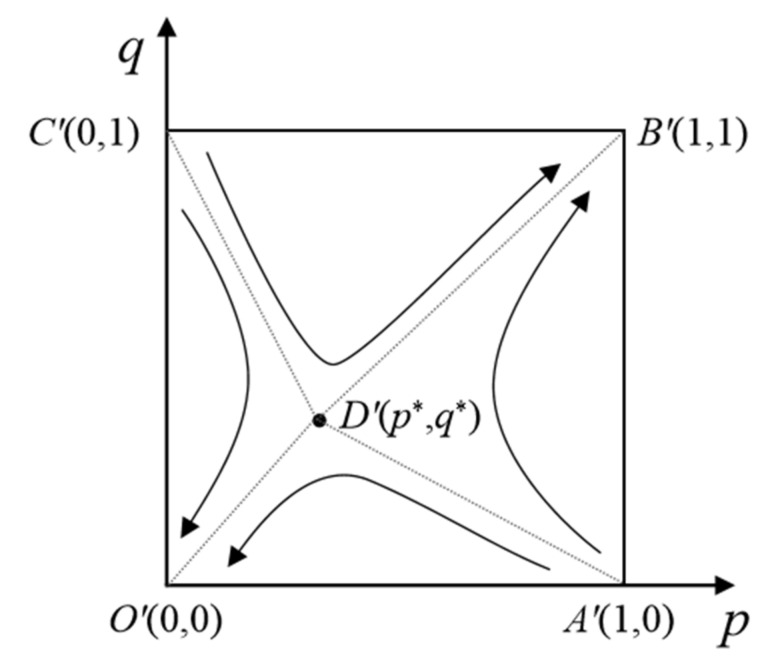
Evolutionary phase diagram of the emergency linkage system.

From the stability theory of differential equations, when $H\left(p\right)=0$ and $H^{\prime }\left(p\right)<0$, *p* is the stability strategy. Similarly, when $K\left(q\right)=0$ and $K^{\prime }\left(q\right)<0$, *q* is the stability strategy. Therefore, when *p* > p*, the equations $K^{\prime }\left(q\right)\left|{}_{q=1}\right.<0$ and $K^{\prime }\left(q\right)\left|{}_{q=0}\right.>0$ are true. At this point, *q* = 1 (positive participation) is the steady state of the game system. On the contrary, when *p* < p*, both $K^{\prime }\left(q\right)\left|{}_{q=1}\right.>0$ and $K^{\prime }\left(q\right)\left|{}_{q=0}\right.<0$ are true. Therefore, *q* = 0 (negative participation) is the steady state of the game system. Consistently, when *q* > q*, *p* = 1 (positive emergency) is the steady state of the game system. When *q* < q*, *p* = 0 (negative emergency) is the steady state of the game system. The corresponding evolution phase diagram of the system was drawn, as shown in Figure 8.

### 5.5. Simulation Analysis of the Emergency Linkage Game Model

In order to observe the strategic behaviors of the local government and social organizations in the emergency linkage game model and to clarify the dynamic evolution mechanism of the emergency linkage game system, a numerical simulation analysis was carried out. The initial values of the simulation parameters were *α* = 0.5, *β* = 0.5, *θ* = 0.5, *ε* = 0.5, *M* = 10, *L*_1_ = 25, *L*_2_ = 10, *T*_1_ = 12, *T*_2_ = 18, *C*_1_ = 15, *C*_2_ = 12, *A*_1_ = 8, and *A*_2_ = 5. Meanwhile, the initial state of the emergency linkage game system was (0.5, 0.5). In the following subsections, the influence of parameters *β*, *ε*, *θ*, *A*_1_, and *A*_2_ on the evolution of game system is investigated in detail.

#### 5.5.1. The Effect of *β* on the Evolution of Emergency Linkage Game System

**Figure 9:**
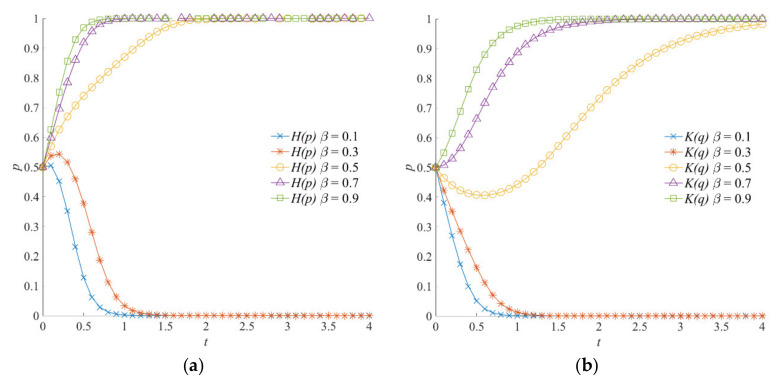
Evolution trajectory of the emergency linkage game system under different values of participation intensity of social organizations. (**a**) The influence of *β* on the evolution of the local government’s strategy. (**b**) The influence of *β* on the evolution of social organizations’ strategies.

The participation intensity of social organizations *β* is a variable parameter describing the initiative of social organizations for participating in natural hazard emergency responses. In the emergency linkage game model, the participation intensity of social organizations *β* is mainly related to the level of social organizations’ perception of natural hazard crisis. The evolution trajectories of the emergency linkage game system under different values of *β* are shown in Figure 9. As the participation intensity of social organizations increases, both the local government and social organizations choose to evolve into positive strategies. On the one hand, when the value of *β* is high, the additional emergency linkage benefits obtained by the local government from social organizations increases, which enhances the willingness of the local government to choose a positive emergency strategy. At the same time, the local government that chooses a positive emergency strategy also drives social organizations to choose positive participation strategies. On the other hand, the emergency capital stock that can be invoked by the local government and social organizations in natural hazard emergency responses and operations is higher, given a high value of *β*. At this time, the offsetting costs of the actual emergency operations are greater. The local government and social organizations are less likely to choose negative strategies.

#### 5.5.2. The Effect of *ε* on the Evolution of the Emergency Linkage Game System

**Figure 10:**
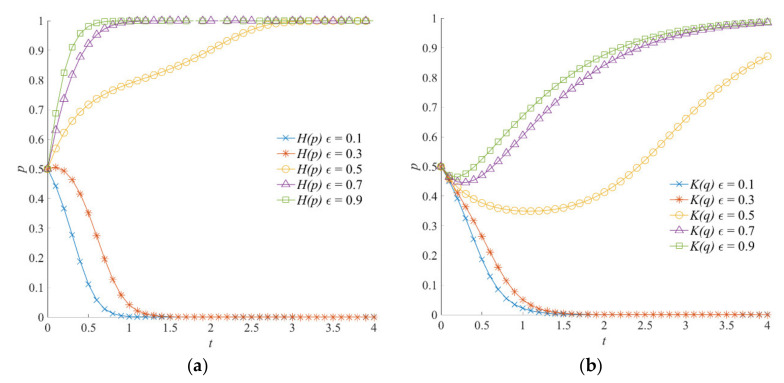
Evolution trajectory of the emergency linkage game system under different coefficients of emergency dispatching. (**a**) The influence of *ε* on the evolution of the local government’s strategy. (**b**) The influence of *ε* on the evolution of social organizations’ strategies.

The coefficient of emergency dispatching *ε* is a variable parameter necessary for measuring the level of the local government’s emergency command. A higher *ε* can effectively avoid the phenomenon of disorderly information gathering, poor information transmission, and blocked connection channels of social organizations and can reduce the cooperation redundancy between local government and social organizations. The evolution results of the emergency linkage game system after simulation, when the coefficient of emergency dispatching *ε* is set as 0.1, 0.3, 0.5, 0.7, and 0.9, are shown in Figure 10. When *ε* = 0.1 or *ε* = 0.3, the negative emergency strategy and negative participation strategy are the stable states of the emergency linkage game system. When ε is 0.5, 0.7, or 0.9, with the increase in evolution time, the emergency linkage game system will converge to (1, 1). With the increase in *ε*, the local government showed a higher tendency to choose a positive emergency strategy. Similarly, with the increase in *ε*, social organizations also tend to choose a positive participation strategy. However, the strategy evolution speed of social organizations is slow—this is because the coefficient of emergency dispatching *ε* has no direct influence on the expected income of social organizations. However, driven by the positive emergency strategy of the local government, social organizations gradually converge to *q* = 1, given the high coefficient of emergency dispatching *ε*.

#### 5.5.3. The Effect of *θ* on the Evolution of the Emergency Linkage Game System

**Figure 11:**
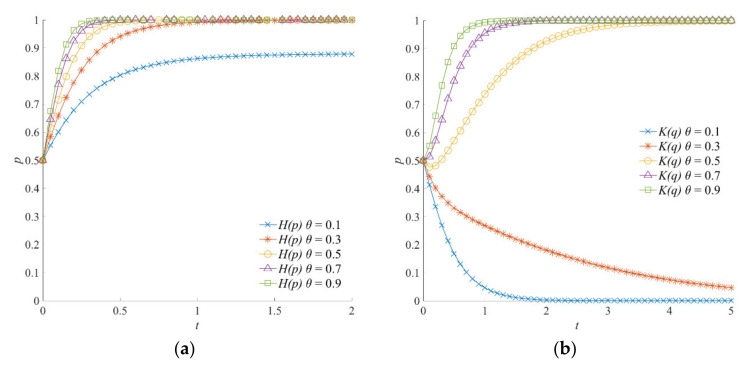
Evolution trajectory of the emergency linkage game system under different degrees of emergency cooperation. (**a**) The influence of *θ* on the evolution of the local government’s strategy. (**b**) The influence of *θ* on the evolution of social organizations’ strategies.

The *θ* is an index variable necessary for evaluating the degree of emergency cooperation between the local government and social organizations. When the degree of emergency cooperation *θ* is high, the local government and social organizations achieve a higher level of information exchange and resource sharing. The evolution trajectories of the emergency linkage game system under different degrees of emergency cooperation *θ* are shown in Figure 11. With the increase in the degree of emergency cooperation *θ*, the perception of losses caused by natural hazards and derivative disasters by the local government and social organizations gradually decreases. For the local government, the probability of selecting a positive emergency strategy gradually increases, and the evolution speed of stable strategy accelerates accordingly. For social organizations, the strategy selection gradually shifts from negative to positive participation. To sum up, in the emergency linkage game system, the strategy combinations of the local government and social organizations change from (1, 0) to (1, 1). Meanwhile, by comparing Figure 11a,b, it can be seen that, when the system moves to (1, 1), the local government reaches a stable state more quickly than social organizations. This indicates that the local government is more direct in strategy selection under different degrees of emergency cooperation *θ*.

#### 5.5.4. The Effect of Emergency Capital Stock on the Evolution of the Emergency Linkage Game System

##### The Emergency Capital Stock *A*_1_

**Figure 12:**
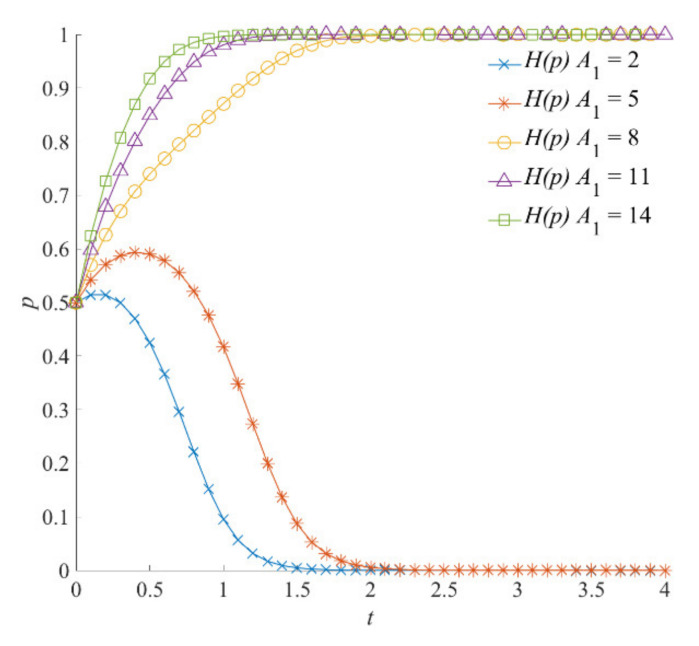
The impact of emergency capital stock *A*_1_ on the evolution of the local government’s strategies.

The results of the impact of emergency capital stock *A*_1_ on the strategy evolution of the local government are shown in Figure 12. When *A*_1_ is high, the local government more effectively implements the preparation of emergency plans and emergency rescue drills in the linkage of emergency preparedness. At this point, higher institutional capital stock and trust capital stock are formed. With the increase in emergency capital stock *A*_1_, the costs for the local government in natural hazard emergency responses and operations decrease accordingly. When *A*_1_ is 8, 11, and 14, the local government’s emergency strategy converges to *p* = 1. At the same time, when *A*_1_ = 2 or *A*_1_ = 5, the emergency capital stock formed by the local government in the linkage of emergency preparedness is insufficient. Although the local government chooses a positive emergency strategy due to the emergency needs and the responsibility requirements in the early stage of evolution, this eventually changes to negative emergency strategy due to the high costs of emergency operations.

##### The Emergency Capital Stock *A*_2_

**Figure 13:**
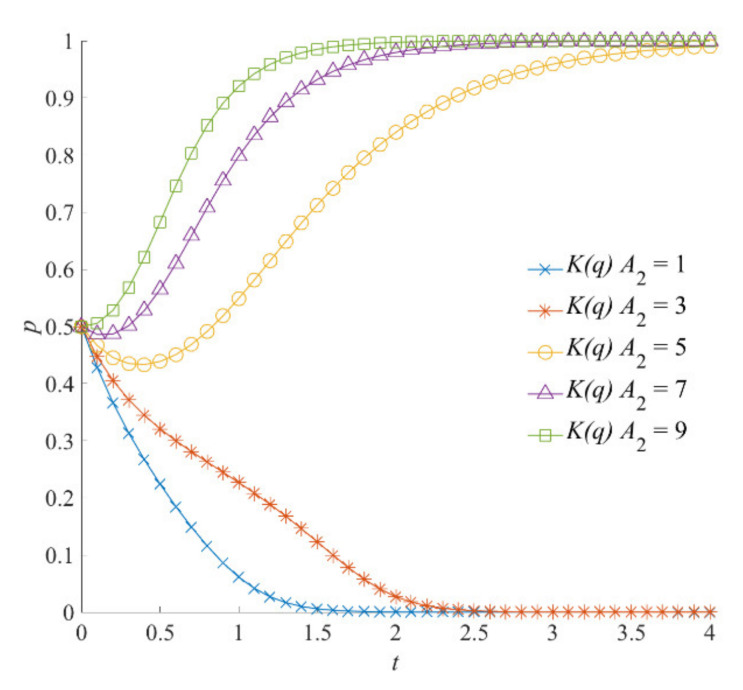
The impact of emergency capital stock *A*_2_ on the evolution of social organizations’ strategies.

Under different values of emergency capital stock *A*_2_, the evolution results of social organizations’ participation strategies are shown in Figure 13. When *A*_2_ = 1 or *A*_2_ = 3, the evolutionarily stable strategy of social organizations is negative participation. With the increase in *A*_2_, the costs of social organizations’ participation in natural hazard emergency responses gradually decrease. The enthusiasm and initiative of social organizations to participate in natural hazard emergency responses effectively improves, and the shift of social organizations to positive participation strategies is further promoted. At the same time, it can be seen that, when *A*_1_ = 5, the evolutionarily stabile strategy of the local government is the negative emergency strategy. However, when *A*_2_ is equal to 5, social organizations shift to the strategy of positive participation. This is because the costs of social organizations’ participation in emergency operations are relatively low. Therefore, a greater proportion of the emergency capital stock formed between the local government and social organizations can cover and offset the costs of emergency operations. It is more straightforward for social organizations to choose positive participation strategies.

## 6. Discussion

The efficiency of natural hazard emergency responses and operations depends on the orderly cooperation of all emergency organizations. The local government and social organizations, as the core organizations in the process of natural hazard emergency responses, have multiple levels and various types of cooperation and interaction. The emergency game model of the social organizations participating in natural hazard responses constructed in this paper effectively describes this problem. At the same time, based on the complementary strategy combination of incentive and linkage, the emergency incentive game model and the emergency linkage game model were set up from the perspective of the local government’s decision making. The influence mechanism of different strategies on the cooperative relationship between the local government and social organizations was discussed, and the practical path to improve the enthusiasm of social organizations for participating in natural hazard emergency responses was explored.

According to the numerical simulation results of the emergency incentive game model and the emergency linkage game model, in order to encourage social organizations to choose positive participation strategies, the local government needs to take the following measures. On the one hand, the local government needs to increase the amount and intensity of compensation and rewards for social organizations. Meanwhile, the scope of recognition for social organizations should also be expanded. On the other hand, the local government should strengthen the level of emergency linkage with social organizations, accumulate the emergency capital stock formed in the stage of emergency preparation, and improve the coefficient of emergency dispatching and the degree of emergency cooperation in the stage of emergency treatment.

From the perspective of the mechanism of the strategy, compensation, economic, and reputation incentives enhance the enthusiasm of social organizations for participating in emergency operations through improving the perception of benefits. The linkage of emergency preparedness and emergency response is to realize the positive participation of social organizations in natural hazard emergency responses by reducing the cost expenditure of emergency operations and disaster loss perception. In the process of emergency preparedness, when the local government and social organizations form an emergency capital stock based on institutional norms and cooperative trust, the emergency costs in emergency operations will effectively reduce, and the tacit cooperation will improve. Therefore, the emergency linkage game system promotes the formation of a positive strategy combination. In the process of emergency response, the local government and the social organizations need to reduce the perception of loss caused by natural hazard and derivative disasters. At this point, the local government and the social organizations improve the matching degree of emergency cooperation to achieve rapid and efficient emergency operations. To some extent, it can reduce the time of emergency disposal and avoid the loss of derivative disasters. Therefore, the emergency linkage strategy reduces the perception of losses between the local government and the social organizations. The lower the perceptions of losses incurred by natural hazards are, the more likely the local government and the social organization choose positive strategies.

As can be seen from Figure 3, Figure 10 and Figure 11, when both the local government and social organizations tend to choose positive strategies, the slope of the evolution trajectory curve of the local government to positive strategy is larger. That is, the convergence rate of the local government to positive emergency strategies is faster. In other words, when social organizations choose positive participation strategies, the strategy selection is relatively lagging behind. Therefore, it is concluded that the local government’s selection of positive emergency strategies is the driving force for the evolution of social organizations to positive participation strategies.

When analyzing the influence of the local government’s incentive intensity *μ* on the evolution of the emergency incentive game system, we can elicit that the value of *μ* has different effects on the local government and social organizations. The will of social organizations choosing positive participation strategies is stronger with a high value of *μ*, but the same is not true for the local government. A larger *μ* value signifies that the incentive costs *μW* are high, costs that the local government needs to pay when implementing various incentive measures. When the incentive cost expenditure cannot bring sufficient benefits of emergency operations and emergency cooperation for the local government, the probability of the local government choosing a positive emergency strategy decreases accordingly. Therefore, with the increase in *μ*, the local government’s strategy selection is expressed as a “negative–positive–negative” fluctuation trend.

In addition, this article still has some limitations in model design and data source. On the one hand, the government and the social organizations are bounded rational in both the emergency incentive game model and the emergency linkage game model. The game subjects’ social preferences are not considered. At the same time, there are many types of governments and social organizations. The different levels of governments and regional governments and different types of social organizations show different behavioral preferences in natural hazard emergency cooperation. The discussion on the heterogeneity of game subjects deserves further study. The strategy selection of the government and social organizations in emergency response is a complex dynamic process. Therefore, it is necessary to optimize and improve the model according to internal and external factors. On the other, numerical simulation is used to analyze the influence mechanism of different parameters on the emergency incentive game system and emergency linkage game system. The initial setting of parameters in the model need to be further optimized. It is necessary to analyze and discuss the behavior evolution of the government and social organizations in natural hazard emergency response based on actual cases.

## 7. Implications

In order to further enhance the level of emergency cooperation between the local government and social organizations and to improve the organizational framework of natural hazard emergency management, combined with the decision-making interaction mechanism and dynamic evolution of the local government and social organizations, we propose the following countermeasures and suggestions in line with the practice of natural hazard emergency management.

(1) Paying attention to the combination of management and service, rewards, and publicity, so as to stimulate the enthusiasm of social organizations to participate in emergency operations. First, governments should improve the reward and compensation mechanisms for social organizations participating in emergency responses. Social organizations participating in emergency operations should be compensated with funds in terms of human costs, organizational costs, and equipment wastage, and compensation clauses of emergency requisition should be formulated. Meanwhile, social organizations that actively participate in emergency operations and perform outstanding tasks should be rewarded. Second, typical deeds of social organizations participating in emergency responses should be widely publicized by means of emergency response summary, public service recognition, and emergency response documentary. Social organizations that play a key role in emergency management should be commended, so as to form a positive public opinion orientation for emergency responses and create a positive atmosphere for the social organizations participating in emergency management. Third, in daily emergency preparedness, the local government should enhance the public’s crisis awareness through activities such as emergency science popularization, emergency publicity and education, and natural hazard census, so as to enhance the participation intensity of social organizations in natural hazard emergency responses.
(2) Forming a coordinated cooperation mechanism for emergency preparedness and responses to improve the efficiency of natural hazard emergency responses. First, governments and their relevant departments should be fully aware of characteristics (i.e., personnel composition, professional characteristics, and modes of action) of the social organizations within their jurisdiction that are willing and able to participate in natural hazard emergency responses. Furthermore, governments and their relevant departments can carry out the daily business guidance and emergency task assignment of social organizations smoothly. Second, specific matters concerning the participation of social organizations in natural hazard emergency responses should be included in the formulation of emergency plans. Meanwhile, governments and social organizations should be integrated to carry out various forms of emergency preparedness, such as emergency drills and joint training, so as to ensure close cooperation and rapid response between governments and social organizations in a natural hazard emergency. Third, it is necessary to strengthen the overall deployment of governments and social organizations in emergency responses. To realize the coordination and unification of various emergency forces is beneficial for reducing the costs of the manpower, organization, and coordination of emergency operations. Then, ineffective emergency command information, incomplete order execution, and other prominent problems can be avoided.
(3) Encouraging the driving role of the local government in a natural hazard emergency and guiding social organizations to actively participate in emergency response operations. First, it is necessary to guide social organizations to participate in emergency responses in an orderly manner, i.e., ensure coordinated action in an organized, divided, and orderly manner and maintain order at the scene of the disaster. Governments should, in light of the actual situation of natural hazards, encourage social organizations to participate in the patrolling of key targets, screening of risks and hidden dangers, evacuation of people, search and rescue of personnel, transportation of supplies, dredging of roads, and voluntary services. Second, governments should strengthen the management of the receipt, allocation, and transshipment of emergency support materials. It is necessary not only to coordinate all kinds of materials raised and provided by foundations, public welfare associations, and other social organizations but also to provide necessary transportation, communications, logistics, equipment, and other support measures to social organizations. Third, relevant functional departments need to improve the policy guarantee system for social organizations to participate in natural hazard emergency responses. On the basis of fully considering the coordination relationship between social organizations and emergency response tasks, the role of social organizations in the emergency response to natural hazards should be clarified. On this basis, the management methods, implementation procedures, and implementation measures for social organizations to participate in natural hazard emergency responses need to be further improved.

## 8. Conclusions

In order to analyze the interaction between the local government and social organizations in natural hazard emergency responses, the incentive and linkage models of social organizations participating in natural hazard emergency responses were constructed based on evolutionary game theory. The interaction between the local government and social organizations was analyzed by numerical simulation, and the influence mechanism of the government’s incentive and linkage strategies on social organizations’ participation in natural hazard emergency responses was discussed. The key research conclusions are as follows:

(1) To improve the emergency efficiency of natural hazards, cooperation between the local government and social organizations should be strengthened. The participation intensity of social organizations, the coefficient of emergency dispatching, and the degree of emergency cooperation and emergency capital stock have positive influences on the local government’s choice of positive strategies. Correspondingly, compensation, reward, reputation, and emergency capital stock have positive effects on social organizations’ choice of positive participation strategies. Therefore, the incentive or linkage strategy adopted by the local government can significantly improve the enthusiasm of social organizations to participate in a natural hazard emergency and can encourage social organizations to choose positive participation strategies.
(2) In an emergency incentive game model, when the incentive intensity of the local government is too high, the local government shifts from a positive to a negative emergency strategy. This shows that overreliance on incentive measures reduces the probability that the local government will choose a positive emergency strategy and will inhibit the formation of a positive strategy combination of the game model.
(3) In the game model of social organizations participating in natural hazard emergency responses, the evolution rate of the local government to a positive emergency strategy is generally faster than that of social organizations. This indicates that the strategy selection of the local government drives that of social organizations.

In this article, the strategy type of the government guiding social organizations to participate in natural hazard emergency response was extended to incentive and linkage, which is of great significance for improving the efficiency of government emergency decision-making and enriching government emergency strategy measures. The government can improve the efficiency of emergency cooperation with social organizations by improving funding and material compensation, economic rewards, reputation benefits, incentive intensity, the coefficient of emergency dispatching, and the degree of emergency cooperation and emergency capital stock. The research conclusion effectively solved the three research problems raised in the introduction. The countermeasures and suggestions put forward have positive reference value for the government’s practice of natural hazard emergency response. Meanwhile, there is still room for further work and improvement on these research results. On the one hand, when analyzing the emergency cooperation between the local government and social organizations in natural hazards, in this paper, we did not subdivide the types of the local government and social organizations. Governments can be divided into emergency forces and security institutions, while social organizations can be divided into professional types, hub types, and public welfare associations. Therefore, the interaction and game relationship between different types of emergency organizations are complex, and it is necessary to further explore the problem in future studies. On the other hand, in this paper, we only considered the game interaction between the local government and social organizations. However, there are still other emergency organizations involved in natural hazard emergency responses. The interaction and game with other emergency organizations may affect the strategy selection of the local government and social organizations; therefore, this aspect needs to be further clarified in future studies.

## Figures and Tables

**Table 1:** Evolutionary game study on social organizations’ participation in emergency operations.

Authors	Emergency Scenario	Game Subject	Influence Factors
Chen, F.; Liu, S.; Appolloni, A. [44]	Humanitarian supply chain in disasters	International nongovernment organizations (INGOs)	The amounts of sharable resources, coordinated costs, coordinated benefits coefficient of resources sharing.
		Local nongovernment organizations (LNGOs)	
Xu, Z.; Cheng, Y.; Yao, S. [45]	Public health emergencies	Local government	Supervision cost, subsidy coefficient, rewards, punishment.
		Enterprises	
		The public	
Du, L.; Qian, L. [46]	Disaster relief	Government	The cooperation benefit, reward incentive, punishment for nonfeasance, efficiency of response, cost of coordination, and value of legitimacy.
		Government-owned nonprofit organizations (GONPOs)	
	Disaster mobilization	Government	
		Grassroots nonprofit organizations (GRNPOs)	
Hou, J.; Lv, J. [47]	The geo-disaster	Government	Excess earnings from cooperation, the cost paid by the sides respectively, the discount factor.
		The nonprofits	

**Table 2:** Definition and ranges of the parameters in the emergency incentive game model.

Symbol	Definition	Ranges
*C*_1_	The costs of the local government’s emergency operations.	*C*_1_ > 0
*C*_2_	The costs of social organizations’ emergency operations.	*C*_2_ > 0
*L*_1_	The local government’s perceptions of losses incurred by natural hazards.	*L*_1_ > 0
*L*_2_	Social organizations’ perceptions of losses incurred by natural hazards.	*L*_2_ > 0
*T*_1_	The emergency benefits obtained by the local government in implementing emergency responses and operations.	*T*_1_ > 0
*T*_2_	The emergency benefits obtained by the local government as a result of the social organizations’ participation in emergency operations.	*T*_2_ > 0
*G*_1_	Compensation of funds and materials given to social organizations by the local government.	*G*_1_ > 0
*G*_2_	Rewards issued by the local government to social organizations for adopting a positive emergency participation strategy.	*G*_2_ > 0
*R*_1_	Social reputation benefits obtained by the local government for implementing emergency operations.	*R*_1_ > 0
*R*_2_	Social reputation benefits obtained by social organizations for implementing emergency operations.	*R*_2_ > 0
*W*	Incentive costs paid by the local government.	*W* > 0
*α*	The emergency response intensity of the local government.	0 ≤ *α* ≤ 1
*β*	The participation intensity of social organizations.	0 ≤ *β* ≤ 1
*μ*	The incentive intensity of the local government.	0 ≤ *μ* ≤ 1

**Table 3:** Payoff matrix of the emergency incentive game model.

Game Subjects and Behavior Strategies		Social Organizations	
		Positive Participation (*y*)	Negative Participation (1 − *y*)
Local government	Positive emergency (*x*)	$-C_{1}-L_{1}-W+T_{1}+R_{1}+T_{2}$, $-C_{2}-L_{2}+G_{1}+G_{2}+R_{2}$	$-C_{1}-L_{1}-W+T_{1}+R_{1}+\beta T_{2}$, $-\beta C_{2}-L_{2}+\beta G_{1}+\beta R_{2}$
	Negative emergency (1 − *x*)	$-\alpha C_{1}-L_{1}-\mu W+\alpha T_{1}+\alpha R_{1}+\mu T_{2}$, $-C_{2}-L_{2}+\mu G_{1}+\mu G_{2}+\mu R_{2}$	$-\alpha C_{1}-L_{1}-\mu W+\alpha T_{1}+\alpha R_{1}+\mu \beta T_{2}$, $-\beta C_{2}-L_{2}+\mu \beta G_{1}+\mu \beta R_{2}$

**Table 4:** Definition and ranges of the parameters in the emergency linkage game model.

Symbol	Definition	Ranges
*C*_1_	The costs of the local government’s emergency operations.	*C*_1_ > 0
*C*_2_	The costs of social organizations’ emergency operations.	*C*_2_ > 0
*L*_1_	The local government’s perceptions of losses incurred by natural hazards.	*L*_1_ > 0
*L*_2_	Social organizations’ perceptions of losses incurred by natural hazards.	*L*_2_ > 0
*T*_1_	The emergency benefits obtained by the local government in implementing emergency responses and operations.	*T*_1_ > 0
*T*_2_	The emergency benefits obtained by the local government as a result of social organizations’ participation in emergency operations.	*T*_2_ > 0
*A*_1_	The emergency capital stock of the local government formed by the linkage of emergency preparedness.	*A*_1_ > 0
*A*_2_	The emergency capital stock of social organizations formed by the linkage of emergency preparedness.	*A*_2_ > 0
*M*	Emergency linkage costs of the local government.	*M* > 0
*α*	The emergency intensity of the local government.	0 ≤ *α* ≤ 1
*β*	The participation intensity of social organizations.	0 ≤ *β* ≤ 1
*ε*	The coefficient of emergency dispatching.	0 ≤ *ε* ≤ 1
*θ*	The degree of emergency cooperation.	0 ≤ *θ* < 1

**Table 5:** Payoff matrix of the emergency linkage game model.

Game Subjects and Behavior Strategies		Social Organizations	
		Positive Participation (*q*)	Negative Participation (1 − *q*)
Local government	Positive emergency (*p*)	$-C_{1}+A_{1}-\left(1-\theta \right)L_{1}-M+\varepsilon T_{1}+\varepsilon T_{2}$, $-C_{2}+A_{2}-\left(1-\theta \right)L_{2}$	$-C_{1}+\beta A_{1}-L_{1}-M+\varepsilon T_{1}+\beta \varepsilon T_{2}$, $-\beta C_{2}+\beta A_{2}-L_{2}$
	Negative emergency (1 − *p*)	$-\alpha C_{1}+\alpha A_{1}-L_{1}-\alpha M+\alpha \varepsilon T_{1}+\alpha \varepsilon T_{2}$, $-C_{2}+\alpha A_{2}-L_{2}$	$-\alpha C_{1}+\alpha \beta A_{1}-L_{1}-\alpha M+\alpha \varepsilon T_{1}+\alpha \beta \varepsilon T_{2}$, $-\beta C_{2}+\alpha \beta A_{2}-L_{2}$

## Data Availability

Not applicable.
